# Supporting self-management and clinic attendance in young adults with type 1 diabetes: development of the D1 Now intervention

**DOI:** 10.1186/s40814-021-00922-z

**Published:** 2021-10-12

**Authors:** Eimear C. Morrissey, Bláthín Casey, Lisa Hynes, The D1 Now Young Adult Panel, Sean F. Dinneen, Molly Byrne

**Affiliations:** 1 Health Behaviour Change Research Group, School of Psychology, National University of Ireland, Galway, Ireland; 2 School of Medicine, National University of Ireland, Galway, Ireland; 3 Health Research Institute, University of Limerick, Limerick, Ireland; 4 Discipline of General Practice, School of Medicine, National University of Ireland, Galway, Ireland

**Keywords:** Type 1 diabetes, Young adulthood, Self-management, Intervention development

## Abstract

**Background:**

Self-management of type 1 diabetes (T1D) is complex and can be particularly challenging for young adults. This is reflected in the high blood glucose values and rates of clinic non-attendance in this group. There is a gap for a theory-based intervention informed by key stakeholder opinions to support and improve self-management in young adults with T1D.

**Purpose:**

The aim of the work was to systematically co-develop an evidence-based and stakeholder-led intervention to support self-management and clinic engagement in young adults living with T1D in Ireland. Co-development was led by the Young Adult Panel.

**Methods:**

The Behaviour Change Wheel was used to guide the development. Five evidence sources were used to inform the process. An iterative co-design process was used with the Young Adult Panel. Initial intervention components were refined and feasibility tested using qualitative methods.

**Results:**

Environmental restructuring, education and training were selected as appropriate intervention functions. The co-design process, along with qualitative refinement and feasibility work, led to the final intervention content which consisted of 17 behaviour change techniques. The final D1 Now intervention consists of three components: a support worker, an agenda setting tool and an interactive messaging service.

**Conclusions:**

The D1 Now intervention is now at pilot evaluation stage. Its transparent and systematic development will facilitate evaluation and future replications.

**Supplementary Information:**

The online version contains supplementary material available at 10.1186/s40814-021-00922-z.

## Key messages regarding feasibility

- In order to develop an effective intervention to improve outcomes in young adults living with type 1 diabetes, we needed to use theory, evidence, stakeholder input and public and patient involvement.
- Findings from a systematic review, an expert meeting and several rounds of qualitative research were used to by the research team and Young Adult Panel to develop and refine an intervention, D1 Now.
- The final D1 Now intervention consists of three components: a support worker, an agenda setting tool and an interactive messaging service. Feasibility and acceptability will be assessed in a pilot randomised controlled trial.

## Introduction

Young adults living with type 1 diabetes (T1D) have been highlighted as being at risk of lower engagement with self-management and higher blood glucose levels in comparison to younger and older people with the condition [1, 2]. Self-management of T1D is complex and requires many daily tasks such as the administration of insulin and monitoring of blood sugars and ketones [3]. It also involves frequent visits to diabetes clinics and retinopathy screening services. Balancing the management of this complex chronic condition with the demands and unpredictability of young adulthood can be challenging [4] and this is seen in the high blood glucose values [2] and descriptions of diabetes distress in this group [5, 6]. There is also a high rate of clinic non-attendance in this cohort, which is problematic as regular clinic attendance is associated with improvements in haemoglobin A1c (HbA1c) and a reduction in associated risks [7]. Good relationships between young adults and service providers are cited as being an important factor in encouraging clinic attendance [7, 8]. A recent systematic review of interventions to improve outcomes in young adults living with T1D found that the quality of reported studies was poor, demonstrating a gap for a theory-based intervention informed by key stakeholder opinions to support and improve self-management in young adults with T1D [9].

Guidance for complex intervention development is now widely available and used, such as the Behaviour Change Wheel (BCW) guide to designing interventions [10]. Complex interventions are often required to address health-related problems and are characterised by multiple interacting components. The BCW outlines systematic and in-depth phases of development work prior to a full trial of a complex intervention. Such a comprehensive approach aims to improve the quality of interventions, maximising the likelihood of interventions being effective and implementable, while also contributing to a cumulative science of behaviour change [11]. The use of theory to understand problems and design interventions is recommended by the BCW.

In addition to the use of theory, the design of complex behavioural interventions should emphasise collaboration with key stakeholders to translate the research into practice [12, 13]. Public and patient involvement (PPI) describes the process of involving members of the public or patient groups in the research process. Involvement can occur at different stages throughout the research process and frameworks exist to support its use [14].

This paper describes the systematic development of an evidence-based and stakeholder-led intervention (called D1 Now) to support self-management and clinic engagement in young adults living with T1D in Ireland. The Behaviour Change Wheel intervention development framework [10] was used to structure the development and incorporate insights from theory, evidence and stakeholders. Our approach to PPI involved establishing a Young Adult Panel (YAP) who have worked with the research team throughout intervention development, piloting, evaluation and implementation stages [15]. The development process is reported using the Guidance for Reporting of Intervention Development (GUIDED) checklist [16] (see Additional file 1: Appendix A) and the intervention is reported using the Template for Intervention Description and Replication (TIDieR) [17] checklist (see Additional file 1: Appendix B).

## Methods

### Intervention context

In Ireland, many hospital outpatient diabetes services offer “young adult” clinics aimed at delivering care to individuals aged approximately 18–25 who have transferred from Paediatric or Transition Clinics [18]. Staff in young adult clinics typically include Consultant Endocrinologists, Diabetes Specialist Nurses, Dieticians and Podiatrists. Clinical Psychology support is lacking in most clinics and extremely limited when available [18]. Young adults are usually offered appointments 3–4 times a year.

### Sources of evidence

The D1 Now team conducted an evidence synthesis and primary qualitative analysis with young adults and healthcare providers (HCPs). We also had formal and informal consultations with policy, practice and researcher groups and frequent consultations with our YAP. These sources of evidence can be seen in Table 1 and are discussed in detail in [4]. We combined findings from these sources in an iterative process to guide each step of the BCW framework and to guide final decision making made by our YAP and interdisciplinary research team.

**Table 1:** Sources of evidence for D1 Now intervention development

Source	Type of activity	Overview of aims and findings	Intervention development phase
‘It makes a difference, coming here’: A qualitative exploration of clinic attendance among young adults with type 1 diabetes [8]	Qualitative research and theory development	• Aimed to develop a theory explaining attendance at a hospital-based young adult diabetes clinic. • Twenty-nine people (21 young adults with type 1 diabetes and eight service providers) from one hospital-based diabetes clinic were interviewed • The importance of building strong relationships between young adults and service providers was a key finding of this work. Collaborative relationships between young adults and service providers increased the perceived value of attendance. Meeting multiple unfamiliar service providers had a negative impact on clinic attendance.	Preliminary evidence building
A systematic review of interventions to improve outcomes for young adults with type 1 diabetes [9]	Systematic review	• Aimed to synthesise the evidence regarding the effectiveness of interventions aimed at improving clinical, behavioural or psychosocial outcomes for young adults with T1D. • Eighteen studies were included and categorised as follows: health services delivery (*n* = 4), group education and peer support (*n* = 6), digital platforms (*n* = 4). • Continuity, support, education and tailoring of interventions to young adults were the most common themes across studies • The effectiveness of these interventions on clinical, behavioural or psychosocial outcomes for young adults with T1D was unclear due to poor reporting and heterogeneity of study design. • There is a lack of high-quality, well designed interventions aimed at improving health outcomes for young adults with T1D. The development of a new intervention is warranted. Possible components to the intervention could be continuity, support, education and tailoring of interventions to young adults.	BCW Phase 1 Steps 1–4
Stakeholder perceptions of barriers & facilitators to self-management among young adults with type 1 diabetes: A qualitative analysis based on the COM-B model [19]	Primary qualitative research	• Aimed to understand the factors which influence young adults diabetes self-management and explore how services and support could be improved. • Semi-structured interviews were conducted with diabetes healthcare providers (*n* = 15) and parents of young adults with T1D (*n* = 10). Three focus groups were conducted with young adults with T1D (*n* = 18). • Thematic analysis was used to analyse the data, which was then further categorised using the framework of the COM-B model to identify the factors that influence self-management. All components of the COM-B model were relevant to self-management but physical and social opportunity factors, such as the influence of service providers seemed to be the dominant drivers. • This study (alongside the systematic review) resulted in three focus areas for an intervention being identified. These were o The way young adults are initially introduced to adult services o Attendance at diabetes clinic appointments and contact between appointments o Building relationships between young adults and service providers • These focus areas went on to form the basis of the consensus meeting.	BCW Phase 1 Steps 1–4
Embedding a user-centred approach in the development of complex behaviour change intervention to improve outcomes for young adults living with type 1 diabetes: The D1 Now study [4]	International expert panel consensus meeting	• Aimed to identify specific strategies to address the three focus areas mentioned above using the Behaviour Change Wheel (Michie et al., 2011). • 18 experts including young adults with T1D, researchers, service providers and policy influencers took part in an Expert Panel meeting. Representative groups were formed and each team was asked to examine two of the three focus areas. The BCW was used to identify specific strategies which could be used. • Seven key areas were identified including (1) strategies to facilitate continuity of care, (2) frequent review of young adult needs, (3) joint engagement of both young adults and service providers in diabetes management, (4) choice around when to transition to adult services, (5) engagement strategies, (6) availability of multiple modes of contact and engagement and (7) a flexible clinic appointment system.	BCW Phase 2 Step 5
Embedding a user-centred approach in the development of complex behaviour change intervention to improve outcomes for young adults living with type 1 diabetes: The D1 Now study [4]	YAP meetings	Aimed to provide insight and information about the intervention context from a patient perspective Aimed to get expert input and guidance into the intervention development, particularly around the operationalisation and mode of delivery of intervention components	BCW Phase 1 Steps 1–4 Phase 2 Step 5 Phase 3 Steps 7 and 8

## Applying the behaviour change wheel approach

The BCW approach (10) includes three phases.

### Phase 1: Understand the behaviour

We used our evidence sources (outlined in Table 1) regarding clinic attendance and self-management to understand and define the problem in behavioural terms [4, 7, 9, 19]. These included a qualitative study that developed a theory of clinic attendance, a systematic review of interventions to improve self-management in young adults with T1D, a qualitative study on the barriers and facilitators to self-management and YAP meetings. Specific barriers and enablers to the targeted young-adult level behaviour and HCP/clinic level behaviours were coded using the COM-B model. This process of early intervention planning has been described in detail in [4].

### Phase 2: Identify intervention options

This was done through an international expert panel consensus meeting (held in June 2016) which aimed to use the BCW [10] to identify specific strategies to address the three focus areas identified in the qualitative research. These were (1) the way young adults are initially introduced to adult services, (2) attendance at diabetes clinic appointments and (3) contact between appointments and building relationships between young adults and service providers. Eighteen experts took part in the Expert Panel meeting. Small teams consisting of individuals from each stakeholder group, including young adults with T1D, researchers, service providers and policy influencers, were formed and each team was asked to examine two of the three focus areas, followed by rounds of discussion with the wider group. Before using the BCW for intervention development, potential strategies for addressing a health problem were explored using a behavioural analysis tool. This tool was used to guide teams to identify and consider strategies for addressing the target areas. Possible strategies were discussed in terms of ability to address focus areas, likelihood of change, practicality and potential for spill over to other behaviours and people. This is described in detail in [4].

### Phase 3: Identify content and implementation options

This was done through YAP meetings and core study team meetings in late 2016 and 2017. Guided by the findings of the evidence sources and the seven key areas identified by the expert panel consensus meeting (see Table 1), possible intervention components and modes of delivery were brainstormed by the research team. These options were then presented to the YAP and international steering committee for feedback to guide final decision making by the D1 Now team. Decision-making was determined by a combination of factors including preferences, experience and expertise, and existing resources, such as funding and time. For example, to address two of the key areas identified during the expert panel meeting—availability of multiple modes of contact and engagement, and flexible clinic set-up—the research team sought to identify or develop a technology-supported solution. Based on guidance from technology experts, developing a new tool or platform would be too costly and time consuming. Given the phase of the research, it was deemed most appropriate to identify an existing tool, which met the intervention aims.

Once components were decided on, they were refined and their feasibility was tested (mainly through qualitative research; see Additional file 1: Appendix C and D) in a cohort of young adults with T1D.

#### Refinement

Refinement took place through interviews and focus groups with 15 young adults living with T1D and 24 HCPs who were recruited through a large diabetes clinic in the West of Ireland in early 2018. These participants were presented with a draft outlining proposed intervention components and asked for their feedback. Interview and focus group feedback was used to identify modifications that might refine the intervention and make it as acceptable, usable and feasible to implement as possible. An iterative approach was taken where data were collected and analysed, refinements made and then further data were collected. Each transcript was then read and re-read and all aspects of the data that identified a barrier or made a suggestion for a possible improvement were tabulated. Possible solutions to the barriers and suggested improvements were presented to the YAP for discussion at monthly YAP meetings during the period of refinement. A modification was implemented if it was deemed acceptable and useful by YAP members. A detailed description of the method can be seen in Additional file 1: Appendix C.

#### Feasibility

Feasibility testing took place with 51 young adults living with T1D and 6 HCPs who were recruited through two diabetes clinics in the West of Ireland in late 2018 and early 2019. These participants who used a refined intervention component over a period of time and were asked about their experience in interviews. Interview transcripts were thematically analysed to identify how the intervention components could be acting as barriers and facilitators to self-management and to inform a logic model of how the active ingredients of the intervention might work. The themes were then reviewed and categorised into the subcategories of the COM-B model. Details on the method of this work can be seen in Additional file 1: Appendix D.

## Results

### Phase 1: Understand the behaviour

As outlined in the introduction, young adults living with T1D are at increased risk of lower engagement with self-management and have high rates of clinic non-attendance. We therefore focused on self-management and clinic attendance behaviours to define the problem in behavioural terms in this intervention development process.

The complexity of self-management of diabetes means that there are a number of individual behaviours involved. In addition, self-management behaviours do not exist in isolation, but rather are closely interconnected. While most are carried out at the young adult level, some exist at the HCP level or can be strongly influenced by HCPs and the health care system. Choosing the modifiable factors to target in order to influence young adult self-management was guided by findings from a qualitative study we conducted, which explored barriers and facilitators to self-management [19]. The qualitative findings from this study are summarised in Table 3. To understand the determinants of young adult self-management, the COM-B model was used as a framework for the qualitative analysis. The COM-B sub-categories, Social Opportunity, Physical Opportunity, Psychological Capability, Reflective Motivation and Automatic Motivation appeared to represent the dominant mechanisms by which self-management was supported or hindered. Specifically, environmental drivers of self-management behaviour, particularly social factors like support and health-related messages, strongly influenced young adults’ capability and motivation to self-manage. This is also in line with the theory of clinic attendance developed by [7] which places the relationship between young adults and their service providers as a key component. Therefore, the D1 Now intervention targets social and physical features of young adult’s self-management environment such as young adults and diabetes service communication, to support self-management and clinic attendance.

### Phase 2: Identify intervention options

During the international expert consensus meeting, seven key areas were identified which included strategies to facilitate continuity of care, frequent review of young adult needs, joint engagement of both young adults and service providers in diabetes management, choice around when to transition to adult services, engagement strategies, availability of multiple modes of contact and engagement and flexible clinic set-up. This process is described in detail in [4]. Reflecting on the qualitative findings and the seven key areas identified by the expert panel, the BCW intervention functions that are most relevant are:

- Young adult level: Environmental restructuring and education
- Healthcare provider level: Environmental restructuring and training

### Phase 3: Identify content and implementation options

We operationalised three intervention components to address the intervention functions, environmental restructuring, education and training. The initial intervention components were the introduction of a key worker, an interactive SMS-based messaging system and an agenda setting tool (Table 2). Mapping of the intervention components to the original Hynes study [9] on barriers and facilitators to diabetes self-management can be seen in Table 3. This table also shows how each of intervention components link back to the COM-B model.

**Table 2:** Initial D1 Now intervention components

Key worker	Interactive SMS-based messaging system	Agenda setting Tool
The key worker in the D1 Now intervention aims to provide continuity and build relationships between the young adult and their healthcare team.	Florence or “Flo” is a software-based SMS text messaging system that presents an easy-to-use friendly interface for patients and clinicians to interact with the aim of assisting diabetes self-management.	The agenda setting tool is used by the young adult before and during consultations. It aims to improve the patient-clinician interaction, enhance shared decision-making and highlight diabetes distress (if present).

**Table 3:** Mapping initial intervention components to the COM-B model and findings from Hynes et al. [19]

COM-B model component		Findings from Hynes et al.	Associated D1 Now intervention component	How the D1 Now intervention component functions?
**Capability**	**Psychological** Young adults are developing cognitive and interpersonal skills for self-management	- Applicable knowledge is the foundation - Capacity to self-regulate and organise	Florence Agenda setting tool Key worker	**Florence:** - Florence can send daily blood glucose reminders and provide feedback on the same. - Florence can also send alcohol safety reminders and reminders to check sick day rules **Agenda setting tool**: - This tool provides a space for the young adult to think about the agenda for their consultation and asks “What would you like to discuss today”. - Allows time to think about possible problems and write them down - avoiding the potential difficulty of verbally expressing problems. **Key worker:** - Will meet the young adult and explore the capacity to self-regulate and reasons for the same through 1:1 discussion. - Will assist the young adult in articulating problems through introduction of the agenda setting tool and in 1:1 discussions. - Will conduct a needs/priority assessment and identify any needs for referral (e.g. structured education)
**Opportunity**	**Social** Communication and relationships are pivotal in supporting the self-management of young adults with T1D **Physical** Design of diabetes clinic can be a significant environmental barrier to young adult self-management	- Self-management expectations are generated from diagnosis - Social influences on adjusting to type 1 diabetes and the role of developmental stages - Engage young adults through personalised education and support - Positive feedback from service providers - Challenge to articulate problems and needs to service providers - Impact of connecting with peers with type 1 diabetes	Key Worker Florence	**Key worker:** - Young adult will build relationship with key worker having moved from paediatric to young adult care. The key worker will conduct needs priority assessments and appropriate referrals. - Can provide link to peer support sessions. **Agenda setting tool:** - This tool provides a space for the young adult to think about the agenda for their consultation and asks “What would you like to discuss today”. - Allows time to think about possible problems and write them down - avoiding the potential difficulty of verbally expressing problems - Provides a section for the young adult and service provider to set goals together **Florence:** - Can send “motivational messages” which link to diabetes support websites and blog posts
		- Challenge of delivering young adult-centred services	Agenda Setting Tool Key Worker	**Agenda setting tool:** - The agenda setting tool aims to build the relationship between the young adult and the healthcare professional during the consultation by allowing the young adult to set own agenda. More holistic discussion also. - The tool has been designed with a cohort of young adults to ensure its young adult centred. - The tool provides a structure to the consultation and ensures the young adults needs are prioritised during the time limited appointment **Key worker:** - The key worker will provide continuity in the clinic and follow-up on any disengagement from the service. They will also aid the young adult in using the agenda setting tool, so the consultations are young adult focused.
Motivation	**Reflective** Reconciling identity and self-management **Automatic** Internal and external factors, including emotions and habits influence self-management	- Resentment versus awareness of self-management benefits - Motivation to demonstrate self-reliance - Reaching a new normal	Florence	**Florence:** - Can send “motivational messages” which have self-affirming messages and links to diabetes blogs and websites **Agenda setting tool** - Provides a section for the young adult and service provider to set goals together **Key worker** - Can work with the young adults to set and track goals and support self-reliance
		- Worry about self-management, diabetes complications and service interactions - Routine supports self-management habits		**Florence:** - Creating a routine and habit through daily blood glucose reminders. Also by providing feedback and re-enforcement. **Agenda setting tool:** - The tool explore diabetes distress using the Diabetes Distress Scale (2) and Diabetes Distress Scale (17). **Key worker** - The key worker will provide continuity in the clinic and reduce stress around clinic engagement.

The refinement work led to changes being made to each of the intervention components to make them more acceptable to young adults with T1D in Ireland (methods can be seen in Additional file 1: Appendix C). Table 4 illustrates the changes made to the intervention components.

**Table 4:** Refinements in the D1 Now intervention components

Issue identified by qualitative research	D1 Now intervention feature addressing this issue
**Florence**	
Some Florence messages need to be edited—language used	Language of some messages changed
Some Florence protocols need to be edited—amount of out of target BG messages to trigger breach message	Protocol changed from one out of target BG message to three
Unclear who sets the target BG level for the YA	All HCPs using the intervention will be asked to set this target in conjunction with the YA
Florence needs to be individualised for every person	This will now be a key role for the support worker
Concern around who would provide clinical oversight of data generated by Florence	This will now be a key role for the support worker
**Agenda Setting Tool**	
Parts of the AST are not needed as the information is already in the medical record	The “clinical notes” section has been removed—AST is now two pages rather than three
The AST is too long	The “clinical notes” section has been removed—AST is now two pages rather than three
The AST does not feel YA focused	Discussion topics added (drugs, college, healthy eating) and some removed
Where should the AST be stored?	Scanned and stored on clinic computer system
**Key worker**	
YAs and HCPs were unsure if the key worker should be internal or external to the clinic	This will be tested in the pilot RCT
The name “keyworker” implies mental health or social worker—“support worker” is preferred	Name of role has now been changed to “support worker”
The support worker should have clinical experience	This is now part of the job description
Different possible backgrounds identified for the support worker	This is reflected in the job description

The feasibility work led to three main themes and several subthemes being identified in the data. The first theme was “Taking action” and contained subthemes “Access to supports”, “Goal-setting” and “Motivation”. The second theme was “Making things easier” with subthemes of “Help with remembering”, “Trying to organise it all” and “Easier to talk”. The final theme was “Communication” and held subthemes of “A different dynamic” and “Social norms”. These subthemes were then mapped to the COM-B model to identify how the intervention components acted as barriers or facilitators to self-management (Table 5). This analysis suggested that all categories of the COM-B model played a role in how the intervention was used. For example, the COM-B model component “psychological capability” characterised as cognitive and interpersonal skills mapped onto the subtheme of “easier to talk” in which young adults spoke about how using the Agenda Setting Tool allowed them to get over the shyness they sometimes experience in the clinic. Any further suggestions for change were considered and implemented where possible. See Additional file 1: Appendix D for details of this work.

**Table 5:** Mapping of themes generated in feasibility work to the COM-B model

Themes from thematic analysis	COM-B model component	Sub-themes that map onto COM-B model components	Example quote
Taking action	Physical opportunity	Access to supports	“We talked about what I had written - refreshing a DAFNE course and also about the sensor … we got information and also information on things coming up” Young Adult—agenda setting tool
Communication Making things easier	Social opportunity	A different dynamic Social norms Trying to organise it all	“I think I had more input into it [clinic appointment] than previously” Young Adult—agenda setting tool “You know just to have that thought this message is not being sent just to me it’s being sent to everyone else too that needs it so I’m not the only one.” Young Adult—Florence “But yeah it was good to know that I could like pass a message on that way or you know or change something small if I needed to” Young Adult—whole intervention (speaking about support worker)
Making things easier	Automatic motivation	Help with remembering	“I think it's [blood glucose check reminder messages] a good idea you know but mostly for newly diagnosed people to remind them …. when it's the right time because at the beginning you might forget.” Young Adult—Whole intervention (speaking about Florence)
Taking action	Reflective motivation	Goal setting Motivation	“It was actually really good, because like, we actually set a goal, for what we wanted to achieve by the end of the discussion, which was great, so I feel like more so now than in other clinics I’ve gone to before, I actually have answers to my questions. And I have goals to set for the future and for future visits and stuff.” Young Adult—Agenda setting tool “It [blood glucose check reminder messages] made me do my tests and injections and even try and have them done before the message would come through. So I found it very useful in regard that it was helping me keep control of my diabetes…” Young Adult- Whole intervention (speaking about Florence) “It was a motivator for some who didn't test.” HCP—whole intervention (speaking about Florence)
Making things easier	Psychological capability	Easier to talk Help with remembering	“Sometimes you’re too shy to ask so it’s nice to just have it on a piece of paper yeah” Young Adult—agenda setting tool “I mean it was easier for us to open conversations. So sometimes we struggle to open conversations.” HCP—agenda setting tool “Yeah if you kind of lost track of time you got the text you know you’d remember to do it [blood glucose check], yeah I found those handy yeah.” Young Adult—whole intervention (speaking about Florence)

## The finalised D1 Now intervention

**Fig. 1:**
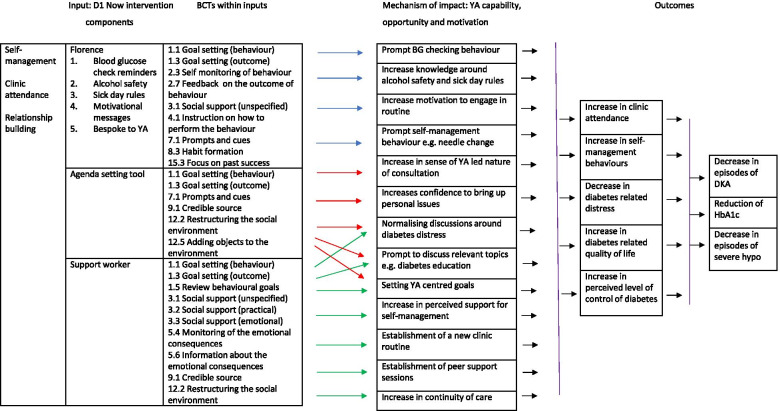
The D1 Now intervention logic model

The D1 Now intervention is described below. Figure 1 depicts the D1 Now logic model as recommended by [20] to articulate and graphically represent the intervention structures, processes and contextual factors intended to achieve the targeted aims and objectives. The behaviour change techniques [21] associated with the finalised intervention are presented within the logic model. There are three primary components in D1 Now: the Support-Worker, the Interactive SMS-based Messaging System and the Agenda Setting Tool. These are detailed further below.

### The support worker

The support worker in the D1 Now intervention aims to provide continuity and build relationships between the young adult and their healthcare team. Briefly, the support worker will attend each young adult clinic appointment and ensure the young adult has set an agenda for their appointment and this agenda is followed through by the healthcare team. This involves screening for diabetes distress using the DDS-2 [22] as part of the agenda setting tool. The support worker will act as an advocate for the young adult on the clinic day and organise a Multidisciplinary Team discussion for each young person (if required) at the end of the clinic. In addition, the support worker will communicate with the young adult between clinic appointments on an individualised basis. Detailed role specifications and duties of the support worker can be found in Additional file 1: Appendix G.

### Interactive SMS-based messaging system

Florence or “Flo” is a software-based SMS text messaging system that presents an easy-to-use friendly interface for patients and clinicians to interact with the aim of assisting diabetes self-management [23]. Text-messaging “protocols” for monitoring a variety of conditions, such as diabetes, chronic obstructive pulmonary disease and respiratory failure have been developed [23, 24]. Our refinement and feasibility work adapted existing diabetes protocols on Florence for an Irish population of young adults with T1D. The system operates by responding to health information sent and received by SMS from the patient.

### The agenda setting tool

**Fig. 2:**
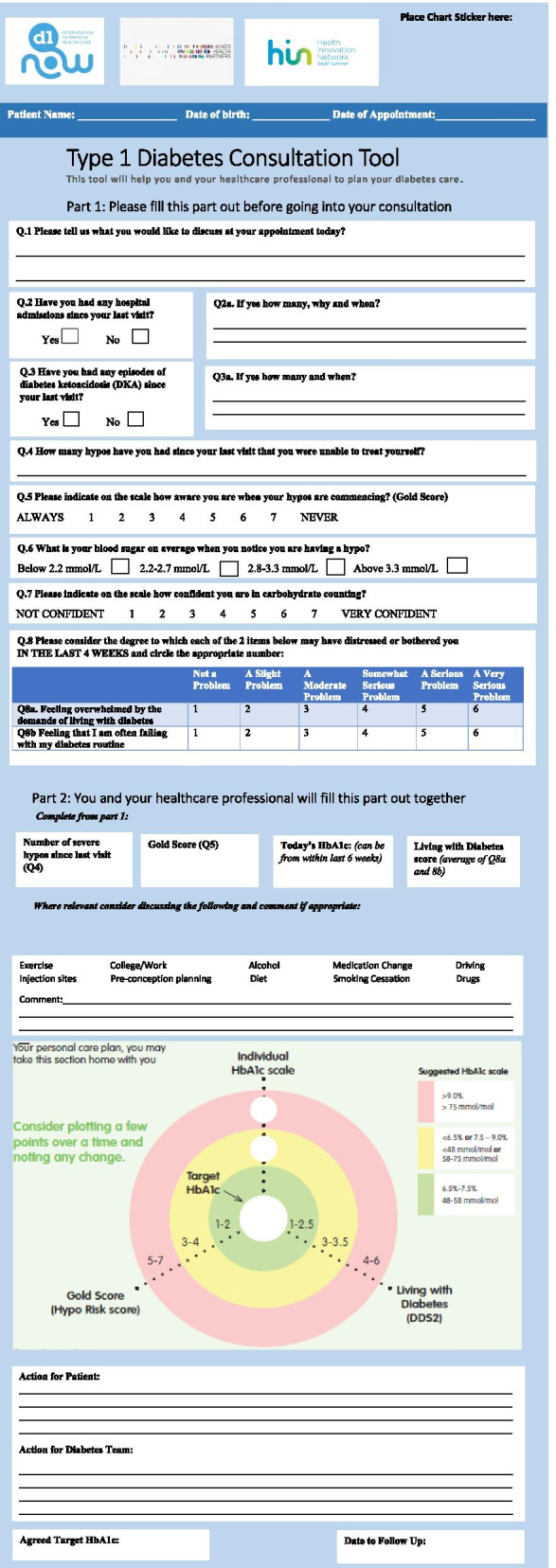
The D1 Now “agenda setting tool”—one of three intervention components

The third intervention component is an agenda setting tool that is used by the young adult before and within consultations and aims to improve the patient-clinician interaction and enhance shared decision-making. Through a scoping review of existing agenda setting tools available internationally, the T1D Consultation Tool (T1C) (Health Innovation Network- https://healthinnovationnetwork.com/projects/type-1-diabetes-consultation-tool-and-user-guide/ ) was chosen for inclusion in D1 Now. The T1C tool is specifically designed for the management of T1D and provides a holistic approach to care planning, bringing together a measure for psychological wellbeing (diabetes distress) as well as clinical results (HbA1c and hypo-unawareness). It enables the clinician to plot the results from the psychological and clinical measures on a dartboard-type chart prompting discussion on the relationship between the three measures (Fig. 2). The tool has 2 parts, the first is completed by the young adult in the waiting room and the second is completed jointly by the young adult and clinician during the consultation. It has been adapted and refined for the Irish young adult context and this can be seen in Fig. 2.

The intervention is described using the TIDieR checklist in Additional file 1: Appendix B.

## Discussion

This paper provides an example of the systematic and structured development of an evidence-based and stakeholder-led intervention to support self-management in young adults with T1D. The intervention is called D1 Now. The paper describes how the BCW was used to comprehensively integrate both evidence and stakeholder perspectives. The intervention development process is reported comprehensively and transparently as this is likely to enhance understanding about the intervention development process [16]. The use of reporting frameworks including GUIDED and TiDIER checklists (Additional file 1: Appendices A and B) enhances the clarity of the intervention development process.

The D1 Now intervention consists of three components: an agenda setting tool, interactive messaging service and support worker, which are based on the intervention functions environmental restructuring, training and education. These functions will be achieved using several BCTs; these are outlined in the logic model in Fig. 2. The self-management of T1D is a challenging process for all living with the condition, and particularly young adults [6]. There are many individual, but interconnected behaviours involved, such as insulin administration, frequent checking of blood glucose levels and managing needle sites [3]. The findings gathered during this programme of research suggested the need for a systematic approach that accounted for relevant developmental and health service factors, found to influence young adult self-management.

Environmental drivers of self-management behaviour, represented by the social and physical opportunity sub-categories of the COM-B model, appeared to strongly influence young adults’ capability and motivation to self-manage. For example, our findings demonstrated the importance of diabetes education, and that the factors acting as barriers to young adults accessing resources like education are system and relationship factors. Similarly, we found that these barriers meant that some young adults find it difficult to broach the topic of diabetes distress. Therefore, the process of intervention development that we engaged in produced intervention components that aim to cultivate ongoing relationships between young adults and the diabetes service to identify and address young adults needs in a timely fashion, and facilitate the development of self-management skills. We hope that the D1 Now intervention will allow for a more holistic diabetes service where the improved relationships will lead to young adults getting both their physical and psychological needs met.

The process of developing a complex intervention needs to be “careful” [25] to prevent inconclusive trial results and research waste. This message is becoming increasingly evident and several sets of guidelines for developing complex interventions have been published to enhance the design of an intervention before examining its effectiveness [11]. A recent attempt to categorise the types of approaches to developing interventions found eight types of approaches with many theories, frameworks and guidelines falling into these categories [26]. While we have used the BCW framework to structure our intervention development, it is possible that other sets of guidelines may also have been beneficial to draw from. We encountered some challenges with the use of the BCW. Self-management of T1D is a complex process consisting of many different behaviours and within many contexts [3]. The BCW method is designed for targeting one behaviour, or a group of similar behaviours. The first stage (steps 1–4) describes “selecting the target behaviour” and “specifying the target behaviour” [10]. This was a challenge in the T1D context, where the behaviours of self-management are linked, and it is not possible or useful to address these behaviours in isolation. As a result, the final intervention is targeted at five of the six components of the COM-B model [10]. The order and timing of the steps also proved a challenge. One of our evidence sources, the qualitative study on stakeholder perceptions of barriers and facilitators to self-management among young adults with T1D, had mapped themes to the COM-B model to identify the drivers of self-management [19]. This piece of work formed the basis for the expert consensus study, where experts used the BCW to generate ideas for intervention functions [4]. Intervention functions were then selected during core team and YAP meetings. We then retrospectively mapped them back to the COM-B model and BCT coded them. While this is not the recommended order of the BCW, it is was pragmatic and best use of available resources at the time. It also ensured that stakeholder involvement was to the fore. In order to ensure the intervention did map to the COM-B model as intended, we used the COM-B model as a framework for analysis of the feasibility work.

The development of the D1 Now intervention involved integrating evidence and stakeholder perspectives. While this allowed a comprehensive approach to intervention development, it was challenging at times to integrate findings from each of these sources. Some of our evidence sources were conducted in parallel (e.g. the systematic review and qualitative study), which meant that findings were combined iteratively throughout the course of intervention development. The international expert consensus meeting was an important step and allowed the development of the intervention to be informed by international expertise and best practice; such that while developed in Ireland, the intervention has global relevance [27]. A strength of our work is the interdisciplinary nature of our team with clinicians familiar with young adult diabetes care working alongside experts in behaviour change. Another strength is the PPI approach reflected in the input from the YAP at all stages of intervention development [15]. When decisions needed to made about which findings to integrate and develop, the expertise and insight of the YAP was invaluable.

### Contribution to future research

The management of T1D during young adulthood is a global issue, with many countries reporting low rates of self-management in this group [2]. While self-management of T1D is complex and consists of a large number of factors, both at the young adult and HCP level, the D1 Now intervention provides an example of an evidence-based approach to addressing some of these factors. If found to be efficacious, it will provide important evidence on supporting self-management in young adults living with T1D. The transparency of the processes of intervention development will allow better testing of the logic model and facilitate future replication or refinement of the intervention [27]. The next stage of the research is to assess the acceptability and feasibility of using the intervention in diabetes clinics as well as the feasibility of running a RCT. A pilot RCT is currently underway to achieve these aims [28].

## Conclusion

This paper provides a rigorous example of the systematic development of an evidence-based and stakeholder-led intervention to support self-management in young adults with T1D. It describes how the BCW was used to comprehensively integrate both evidence and stakeholder perspectives.

## Supplementary Information

**Additional file 1: Appendices**

## Data Availability

The qualitative data that support the findings of this study are available on request from the corresponding author. The data are not publicly available due to privacy restrictions.

## Abbreviations

T1D: Type 1 diabetes
HbA1c: Haemoglobin A1c
PPI: Public and patient involvement
YAP: Young Adult Panel
BCW: Behaviour change wheel
COM-B model: Capability, opportunity, and motivation – behaviour model
BCT: Behaviour change technique
RCT: Randomised controlled trial
HCPs: Healthcare providers
AST: Agenda setting tool
DDS-2: Diabetes distress scale – 2 item
GUIDED checklist: Guidance for Reporting of Intervention Development checklist
TIDieR: Template for Intervention Description and Replication
