# Supplementary material for: Supporting self-management and clinic attendance in young adults with type 1 diabetes: development of the D1 Now intervention
Source: Pilot Feasibility Stud. 2021 Oct 12;7:186. doi: 10.1186/s40814-021-00922-z (PMC8513171; doi:10.1186/s40814-021-00922-z)
Supplement: Supplementary file 1 — Additional file 1: Appendices [file 40814_2021_922_MOESM1_ESM.docx]

Appendix A – GUIDED checklist

| Item description | Explanation | Page in manuscript | Other* |
| --- | --- | --- | --- |
| 1. Report the context for which the intervention was developed. | Understanding the context in which an intervention was developed informs readers about the suitability and transferability of the intervention to the context in which they are considering evaluating, adapting or using the intervention. Context here can include place, organisational and wider sociopolitical factors that may influence the development and/or delivery of the intervention | 3 |  |
| 2. Report the purpose of the intervention development process. | Clearly describing the purpose of the intervention specifies what it sets out to achieve. The purpose may be informed by research priorities, for example those identified in systematic reviews, evidence gaps set out in practice guidance such as The National Institute for Health and Care Excellence or specific prioritisation exercises such as those undertaken with patients and practitioners through the James Lind Alliance. | 2 & 3 |  |
| 3. Report the target population for the intervention development process. | The target population is the population that will potentially benefit from the intervention – this may include patients, clinicians, and/or members of the public. If the target population is clearly described then readers will be able to understand the relevance of the intervention to their own research or practice. Health inequalities, gender and ethnicity are features of the target population that may be relevant to intervention development processes. | 2 |  |
| 4. Report how any published intervention development approach contributed to the development process | Many formal intervention development approaches exist and are used to guide the intervention development process (e.g. 6Squid (16) or The Person Based Approach to Intervention Development (17)). Where a formal intervention development approach is used, it is helpful to describe the process that was followed, including any deviations. More general approaches to intervention development also exist and have been categorised as follows (3):- Target Population-centred intervention development; evidence and theory-based intervention development; partnership intervention development; implementation-based intervention development; efficacy based intervention development; step or phased-based intervention development; and intervention-specific intervention development (3). These approaches do not always have specific guidance that describe their use. Nevertheless, it is helpful to give a rich description of how any published approach was operationalised | 2 |  |
| 5. Report how evidence from different sources informed the intervention development process. | Intervention development is often based on published evidence and/or primary data that has been collected to inform the intervention development process. It is useful to describe and reference all forms of evidence and data that have informed the development of the intervention because evidence bases can change rapidly, and to explain the manner in which the evidence and/or data was used. Understanding what evidence was and was not available at the time of intervention development can help readers to assess transferability to their current situation. | 3, 4 & 5 |  |
| 6. Report how/if published theory informed the intervention development process. | Reporting whether and how theory informed the intervention development process aids the reader’s understanding of the theoretical rationale that underpins the intervention. Though not mentioned in the e-Delphi or consensus meeting, it became increasingly apparent through the development of our guidance that this theory item could relate to either existing published theory or programme theory | 4 |  |
| 7. Report any use of components from an existing intervention in the current intervention development process. | Some interventions are developed with components that have been adopted from existing interventions. Clearly identifying components that have been adopted or adapted and acknowledging their original source helps the reader to understand and distinguish between the novel and adopted components of the new intervention. | 19 |  |
| 8. Report any guiding principles, people or factors that were prioritised when making decisions during the intervention development process. | Reporting any guiding principles that governed the development of the application helps the reader to understand the authors’ reasoning behind the decisions that were made. These could include the examples of particular populations who views are being considered when designing the intervention, the modality that is viewed as being most appropriate, design features considered important for the target population, or the potential for the intervention to be scaled up. | 7 |  |
| 9. Report how stakeholders contributed to the intervention development process. | Potential stakeholders can include patient and community representatives, local and national policy makers, health care providers and those paying for or commissioning health care. Each of these groups may influence the intervention development process in different ways. Specifying how differing groups of stakeholders contributed to the intervention development process helps the reader to understand how stakeholders were involved and the degree of influence they had on the overall process. Further detail on how to integrate stakeholder contributions within intervention reporting are available. | Throughout manuscript |  |
| 10. Report how the intervention changed in content and format from the start of the intervention development process. | Intervention development is frequently an iterative process. The conclusion of the initial phase of intervention development does not necessarily mean that all uncertainties have been addressed. It is helpful to list remaining uncertainties such as the intervention intensity, mode of delivery, materials, procedures, or type of location that the intervention is most suitable for. This can guide other researchers to potential future areas of research and practitioners about uncertainties relevant to their healthcare context. | 14 |  |
| 11. Report any changes to interventions required or likely to be required for subgroups | Specifying any changes that the intervention development team perceive are required for the intervention to be delivered or tailored to specific sub groups enables readers to understand the applicability of the intervention to their target population or context. These changes could include changes to personnel delivering the intervention, to the content of the intervention, or to the mode of delivery of the intervention. | N/A |  |
| 12. Report important uncertainties at the end of the intervention development process. | Intervention development is frequently an iterative process. The conclusion of the initial phase of intervention development does not necessarily mean that all uncertainties have been addressed. It is helpful to list remaining uncertainties such as the intervention intensity, mode of delivery, materials, procedures, or type of location that the intervention is most suitable for. This can guide other researchers to potential future areas of research and practitioners about uncertainties relevant to their healthcare context. | 18&19 |  |
| 13. Follow TIDieR guidance when describing the developed intervention. | Interventions have been poorly reported for a number of years. In response to this, internationally recognized guidance has been published to support the high quality reporting of health care? Interventions and public health interventions. This guidance should therefore be followed when describing a developed intervention. | Appendix 2 |  |
| 14. Report the intervention development process in an open access format. | Unless reports of intervention development are available people considering using an intervention cannot understand the process that was undertaken and make a judgement about its appropriateness to their context. It also limits cumulative learning about intervention development methodology and observed consequences at later evaluation, translation and implementation stages. Reporting intervention development in an open access (Gold or Green) publishing format increases the accessibility and visibility of intervention development research and makes it more likely to be read and used. Potential platforms for open access publication of intervention development include open access journal publications, freely accessible funder reports or a study web-page that details the intervention development process | TBC |  |

Appendix B – TIDieR checklist

| **Item number** | **Item** |  |
| --- | --- | --- |
|  | **BRIEF NAME** |  |
| **1.** | Provide the name or a phrase that describes the intervention. | D1 Now |
|  | **WHY** |  |
| **2.** | Describe any rationale, theory, or goal of the elements essential to the intervention. | The D1 Now intervention aims to support self-management and clinic attendance in young adults (aged between 18-25) with type 1 diabetes (T1D) |
|  | **WHAT** |  |
| **3.** | Materials: Describe any physical or informational materials used in the intervention, including those provided to participants or used in intervention delivery or in training of intervention providers. Provide information on where the materials can be accessed (e.g. online appendix, URL). | Training manual for clinic staff – Appendix E  The Agenda Setting Tool – intervention component (see Figure 2 in manuscript) |
| **4.** | Procedures: Describe each of the procedures, activities, and/or processes used in the intervention, including any enabling or support activities. | The D1 Now intervention consists of 3 components.  *The Support-Worker*  The Support Worker in the D1 Now intervention aims to provide continuity and build relationships between the young adult and their healthcare team. Briefly, the Support Worker, will attend each young adult clinic appointment and ensure the young adult has set an agenda for their appointment and this agenda is followed through by the healthcare team. This involves screening for diabetes distress using the DDS-2 as part of the agenda setting tool. The Support Worker will act as an advocate for the young adult on the clinic day and organise a Multidisciplinary Team (MDT) discussion for each young person at the end of the clinic. In addition, the Support Worker will communicate with the young adult between clinic appointments on an individualised basis.  *Interactive SMS-based Messaging System*  Florence or “Flo” is a software-based SMS text messaging system that presents an easy-to-use friendly interface for patients and clinicians to interact with the aim of assisting diabetes self-management (Cottrell et al, 2012). Text-messaging ‘protocols’ for monitoring a variety of conditions, such as diabetes, chronic obstructive pulmonary disease (COPD) and respiratory failure have been developed (Cottrell et al, 2012., Irwin and De, 2016). The D1 Now study team have adapted existing diabetes protocols on Florence for an Irish population of young adults with T1D. The system operates by responding to health information sent and received by SMS from the patient.  *The Agenda Setting Tool*  The third intervention component is an agenda setting tool that is used by the young adult before and within consultations and aims to improve the patient-clinician interaction and enhance shared decision-making. Through a scoping review of existing agenda setting tools available internationally, the T1D Consultation Tool (T1C) (Health Innovation Network- <https://healthinnovationnetwork.com/projects/type-1-diabetes-consultation-tool-and-user-guide/> ) was chosen for inclusion in D1 Now. The T1C tool is specifically designed for the management of T1D and provides a holistic approach to care planning, bringing together a measure for psychological wellbeing (diabetes distress) as well as clinical results (HbA1c and hypo-unawareness). It enables the clinician to plot the results from the psychological and clinical measures on a dartboard-type chart prompting discussion on the relationship between the three measures. The tool has 2 parts, the first is completed by the young adult in the waiting room and the second is completed jointly by the young adult and clinician during the consultation. It has been adapted and refined for the Irish young adult context and this can be seen in Figure 2 in the main manuscript. |
|  | **WHO PROVIDED** |  |
| **5.** | For each category of intervention provider (e.g. psychologist, nursing assistant), describe their expertise, background and any specific training given. | Support worker – see Appendix F for job spec |
|  | **HOW** |  |
| **6.** | Describe the modes of delivery (e.g. face-to-face or by some other mechanism, such as internet or telephone) of the intervention and whether it was provided individually or in a group. | Support worker: face to face during clinic visits. Online or telephone in between clinic visits  Agenda setting tool: Given by the support worker at clinic visits  Florence: Text messages |
|  | **WHERE** |  |
| **7.** | Describe the type(s) of location(s) where the intervention occurred, including any necessary infrastructure or relevant features. | Young adult diabetes clinics |
|  | **WHEN and HOW MUCH** |  |
| **8.** | Describe the number of times the intervention was delivered and over what period of time including the number of sessions, their schedule, and their duration, intensity or dose. | *First Clinic*  At the first clinic appointment, the young adult will meet with the Support Worker. The Support Worker will introduce themselves and outline their role for the duration of the study period. The Support Worker will then introduce **the agenda setting tool** and ask the participant to complete part 1 of the tool. This includes screening for diabetes distress using the DDS-2 as part of the agenda setting tool. After this, the young adult will meet the team and have their consultation using the agenda setting tool. Once the consultation is complete, before leaving, the young adult will meet with the Support Worker who will ask them how their appointment went, provide them with a copy of their agenda setting tool **and set them up on Florence.**  The Support Worker will individualise the Florence set-up/protocols for each young adult, based on how many messages they want to receive and what targets they want to set. Patients can choose to opt in or opt out of Florence at any time.  At the end of clinic 1, the Support Worker will discuss any need for follow-up before the next clinic appointment. This follow-up can be face-to-face, a phone-call or email in between appointments. The purpose of this follow-up is to 1) check the use of Florence, 2) monitor any actions for the young adult based on their latest appointment, 3) remind/change the next clinic appointment if required and 4) improve engagement. The clinic will finish with a MDT meeting wherein the Support Worker alongside the diabetes team will discuss each young adult and their care needs.  *Clinics 2 and 3*  Subsequent clinic appointments will follow the same procedures. The young adult will first meet the Support Worker and complete the agenda setting tool. The young adult will meet with the rest of the team and complete part 2 of the agenda setting tool and discuss Florence data. The appointment will finish with meeting the Support Worker again and reviewing Florence and any need for follow-up before the next appointment. A MDT meeting will take place. The 3^rd^ clinic appointment will see the Support Worker discharge the young adult from their care and handover to the team through the final MDT meeting |
|  | **TAILORING** |  |
| **9.** | If the intervention was planned to be personalised, titrated or adapted, then describe what, why, when, and how. | One of the intervention components – Florence – is intended to be tailored to each young adult. This issue arose from our qualitative research where every young adult wanted a different amount or type of text message (see Table 4 in main manuscript). When the young adult is setting up Florence with the Support Worker they can chose they type or protocols they want to receive as well as the frequency and timing of these. If they want to change a protocol they can contact the Support Worker who will do so. The Support Worker will also check at each clinic appointment if they are happy with the current protocols or want to change. |
|  | **MODIFICATIONS** |  |
| **10.^ǂ^** | If the intervention was modified during the course of the study, describe the changes (what, why, when, and how). | NA (intervention not yet delivered) |
|  | **HOW WELL** |  |
| **11.** | Planned: If intervention adherence or fidelity was assessed, describe how and by whom, and if any strategies were used to maintain or improve fidelity, describe them. | We are planning to assess fidelity during the pilot RCT. Staff and support worker fidelity will be checked via checklists. Intervention delivery will be assessed via young adult and support worker checklists. |
| **12.** | Actual: If intervention adherence or fidelity was assessed, describe the extent to which the intervention was delivered as planned. | NA (intervention not yet delivered) |

Appendix C – Refinement work

**Aim**

The aim of this refinement work is to refine the components of the D1 Now intervention.

This study uses qualitative methodology to explore the refinement of each of the intervention components by interviewing young adults with T1D and diabetes related Healthcare Professionals (HCPs).

**Participants**

***Young Adults with Diabetes***

As outlined in Table 1, approximately 15 young adults with T1D were recruited. Patients were recruited through [redacted for peer review] where they were identified through a clinical database using the following inclusion/exclusion criteria:

Inclusion:

- Young adults aged between 18 years and 25 years (at time of recruitment).
- Definite diagnosis of Type 1 Diabetes.

Exclusion:

- Attending the paediatric diabetes service.
- Participants with Type 2 Diabetes.

Once identified, potential participants were sent an information letter inviting them to take part in the research study. If interested, participants were then advised to contact the research team directly for further information and were screened for eligibility. Informed written consent was received before participation and participants were allocated to a focus group (as described below).

***Healthcare Professionals (HCPs)***

| **Agenda Setting Tool** |  |
| --- | --- |
| *Young adults n=4* |  |
| Gender | 3 female, 1 male |
| Age | 22-25 (M = 23.5) |
| *HCPs n=6* |  |
| Gender | 5 female, 1 male |
| Occupation | 3 diabetes nurses, 2 dieticians, 1 endocrinologist |
|  |  |
| **Florence** |  |
| *Young adults n=4* |  |
| Gender | 2 female, 2 male |
| Age | 22-25 (M = 23.5) |
| *HCPs n=6* |  |
| Gender | 5 female, 1 male |
| Age | 2 diabetes nurses, 2 dieticians, 2 endocrinologists |
|  |  |
| **Support worker** |  |
| *Young adults n=4* |  |
| Gender | 3 male, 1 female |
| Age | 18-25 (M = 23) |
| *HCPs n=24* |  |
| Gender | 14 female, 10 male |
| Occupation | 2 diabetes nurses, 3 staff nurses, 1 dietician, 1 clinic administrator, 2 biochemists, 1 healthcare assistant, 2 research fellows, 9 junior doctors, 3 endocrinologists |

Twenty four HCPs were recruited using a purposive sampling strategy. This strategy was used to ensure a range of professions and seniority were represented including Consultant Endocrinologists, trainee Endocrinologists, diabetes specialist nurses, pharmacists, psychologists, occupational therapists, administrative staff, etc. HCPs were recruited through staff mailing lists. Once recruited, informed written consent was received.

Table 1. Participant characteristics

**Design**

All participants were presented with the **proposed** intervention component and asked their thoughts and views on it. Participant breakdown and data collection strategy can be seen in Table 2.

Table 2. Participant breakdown by intervention component

| Interactive messaging system | Agenda setting tool | Key worker |
| --- | --- | --- |
| HCP interviews (n = 6)  Young adult focus group (n = 5) | HCP interviews (n = 6)  Young adult focus group (n = 5) | Town Hall meeting with HCPS (n = 24)  Young adult focus groups (n = 5) |

**Analysis**

All interviews and focus groups were transcribed verbatim. NVivo 11 was used to organise the data. Interview and focus group feedback was used to identify modifications that might refine the intervention and make it as acceptable, usable and feasible to implement as possible. An iterative approach was taken where data were collected, analysed, refinements made and then further data collected. Saturation was deemed achieved when no new suggestions or concerns were raised by participants.

The analysis was carried out by a qualitative researcher and an experienced intervention developer. The researchers re-familiarised themselves with the data by reading and re-reading the transcripts. Each transcript was then worked through and all aspects of the data that identified a barrier or made a suggestion for a possible improvement were tabulated. Possible solutions to the barriers and suggested improvements were then brought to the Young Adult Panel (YAP). Monthly YAP meetings during the period of refinement were based around discussions of modifications. A modification was implemented if it was deemed acceptable and useful by YAP members.

**Ethics**

Ethical approval for this study was sought and obtained from [redacted for peer review].

**Appendix D - Feasibility work**

The aim of the feasibility study is assess the feasibility of the D1 Now intervention components, both as lone components and as a package. The data will be mapped to the COM-B model to identify how the intervention components act as barriers and facilitators to self-management.

**Participants**

*Young Adults with Diabetes*

51 young adults with T1D were recruited using similar methods to those described in Appendix A.

*Healthcare Professionals (HCPs)*

Six HCPs were recruited using a pragmatic sampling strategy. This strategy was used to ensure that any HCP who came into contact with any of the intervention components was asked to take part. Once recruited, informed written consent was received.

Table 1. Participant characteristics

| **Agenda Setting Tool** |  |
| --- | --- |
| *Young adults n=44* |  |
| Gender | 27 female, 17 male |
| Age | 18-28 (M = 22) |
| *HCPs n=4* |  |
| Gender | 2 male, 2 female |
| Occupation | 2 consultant endocrinologists, 2 diabetes nurses |
|  |  |
| **Florence** |  |
| *Young adults n=3* |  |
| Gender | 2 female, 1 male |
| Age | 21-24 (M = 22) |
|  |  |
| **Whole intervention** |  |
| *Young adults n=4* |  |
| Gender | 3 male, 1 female |
| Age | 18-29 (M = 23) |
| *HCPs n=2* |  |
| Gender | 2 female |
| Occupation | Diabetes nurse, dietician |

**Design**

All participants **used the intervention components and were retrospectively asked** about their experience of using it.

Table 2. Participant breakdown by intervention component

| Agenda setting tool | Florence | Support Worker | Whole intervention package |
| --- | --- | --- | --- |
| Interviews with young adults (n=44)  Focus group with HCPs  (n=4) | Interviews with young adults  (n=7) | Not feasible to explore due to resource constraints | Interviews with young adults  (n=4)  Joint interview with HCPs  (n=2) |

Feasibility of the Agenda Setting Tool

44 young adults living with T1D used the Agenda Setting Tool as part of their routine young adult clinic appointment at West of Ireland Diabetes Clinic. After their clinic appointment they took part in a brief interview with a member of the research team in the clinic. This took place over four clinic days. An MSc student led the interviews and was supported by two postdoctoral researchers.

4 HCPs who had worked at these clinics and used the Agenda Setting Tool with young adults took part in a focus group. One postdoctoral researcher led the focus group and was supported by another postdoctoral researcher who acted as a moderator for the session.

Feasibility of Florence

7 young adults living with T1D were recruited to use Florence over 4 weeks. To initiate the pilot the young adults were asked to complete a set-up questionnaire so that the SMS messages could be individualized to their requirements for blood-glucose monitoring and alcohol use (as appropriate). The participant was then registered as a user and an account was activated. Participants had to opt into Florence by replying to an ‘opt in’ message with ‘Y’ or ‘N’. All participants who did not have text messages as part of their mobile payment plan were remotely topped up with €20 by the research study team to ensure that engagement with Florence was not impacted by cost. Clinical monitoring was not active for this pilot. Following the 4-week pilot, individual telephone interviews were conducted by an MSc student. Data were collected from 3 pilot participants. Of 7 young adults recruited, 1 never opted in to Florence, 1 opted out after 2 days, 1 engaged only for the first 2 weeks but did not opt out, citing personal reasons for disengagement, 1 completed 100% of the trial but was not available for debrief though researchers made a number of attempts.

Feasibility of the Support Worker

Due to resource constraints it was not feasible to explore the feasibility of the Support Worker as a lone component. This was assessed as part of the whole intervention package instead.

Feasibility of D1 Now

Five young adults living with T1D used the whole intervention package over 12 weeks. This involved using the Agenda Setting Tool in 1-2 clinic appointments. They were also set-up on Florence using the same procedures as above and put on a four weeks on – four weeks off – four weeks on protocol. For the purposes of the feasibility research a member of the research team, who is also trained as a healthcare provider, acted as the Support Worker. Young adults met the Support Worker at any clinic appointments they had over the twelve weeks. They also had telephone contact between appointments. Following the 12 weeks, a postdoctoral researcher conducted telephone interviews with four of the YAs. One YA dropped out due to personal reasons.

Two HCPs had worked in clinic with these YAs and were interviewed after the 12 weeks by a postdoctoral researcher.

**Analysis**

All interviews and focus groups were transcribed verbatim. NVivo 11 was used to organise the data. The five stages of thematic analysis (familiarisation, generation of codes, searching for themes, reviewing themes and defining themes) were followed (Braun and Clarke, 2012). To ensure rigour, coding was partially done by three other researchers and the preliminary themes were discussed at a Young Adult Panel meeting. To identify how the intervention components could be acting as barriers and facilitators to self-management and to inform the logic model, the themes were then reviewed and categorised into the subcategories of the COM-B model by one author. Agreement in terms of the fit of the data within the sub-categories of the COM-B model was assessed by comparing agreement between the lead coder and an expert in the use of these models.

**Ethics**

Ethical approval for this study was sought and obtained from [redacted for peer review].

Appendix E – Training manual for clinic staff

Uploaded as PDF

Appendix F– Job spec for D1 Now Support Worker

**Job Description**

The Support Worker role is an expansion of selected Diabetes teams in the greater Dublin area. The Support Worker has a specific focus on supporting young adults with Type 1 Diabetes and will act as an advocate for these young adults to ensure optimal engagement with the Diabetes service. The successful candidate will work closely with the young adults and their respective Diabetes team (doctors, nurses, allied health care professionals, psychology, etc.).

**Duties/ Key Responsibilities**

- To work closely with the chosen Diabetes sites in the greater Dublin area and members of the D1 Now research team.
- To be a voice/advocate for the young adult through attendance at multidisciplinary meetings within the Diabetes clinic.
- To provide consistency for the young adult by regular attendance at clinics and having a well-rounded knowledge of the young adults medical and social history.
- To communicate with the young adult between their clinical appointments to follow-up on any tasks required and monitor progress with self-management strategies/techniques.
- To manage expectations of the Diabetes service and to conduct a needs/priority assessment for the young adult.
- To assist in organising/rescheduling appointments at the clinic.
- To refer the young adult to structured education, psychology or other services within the diabetes clinic though communication with the multidisciplinary team.
- To work closely with the D1-Now intervention team and additional D1 Now components including an interactive online tool for self-management and an agenda setting tool for use in clinic appointments.
- To be flexible in communicating with the young adult in a suitable location (hospital or community setting) and in a suitable medium (face-face meeting, phone, text, group, etc.)
- To aid relationship building between the young adult and their Diabetes team.
- To carry out administrative duties as required by the post.
- To share information with the Diabetes team and D1-Now team and other services as appropriate in accordance with good practice.
- To be flexible regarding working hours in line with the needs of the role (incorporating occasional late evening work, etc.).
- To participate in education, training and networking events as appropriate

**Qualifications/Skills Required**

**Essential Requirements**

- A recognised professional qualification in Nursing, Mental Health Nursing, Psychology* (postgraduate qualification in Clinical/Counselling/Educational Psychology/Health Psychology), Social Work or Allied Health Care.

*Assistant Psychology posts will be provided supervision by a Clinical Psychologist.

- Experience of working with clinical populations, ideally young people or people with long-term conditions, in a clinical setting (hospital or community).
- Well-developed communication skills and an ability to interact with both young people and adults on a one to one basis and in small groups, within a range of contexts.
- Confident lone working with the young adult patient.
- Ability to plan and manage own workload.
- Highly motivated and a passion for working with young adults.
- Willing to be flexible and work evenings where required.
- Proficiency with computer systems.

**Desirable Requirements:**

- Experience of working with people with Diabetes or young adults with long-term conditions.
- An understanding of diabetes and the issues faced by young people as they transition to adult services.
- Knowledge and understanding of the physical, social and emotional developmental needs of young people.
- Experience of working in a multidisciplinary team in the hospital setting in Ireland.
- Some knowledge and/or experience of motivational interviewing or appropriate behavioural training.
